# The Role of Genetic Resistance in Rice Disease Management

**DOI:** 10.3390/ijms26030956

**Published:** 2025-01-23

**Authors:** Andrews Danso Ofori, Tengda Zheng, John Kwame Titriku, Charlotte Appiah, Xing Xiang, Abdul Ghani Kandhro, Muhammad Irfan Ahmed, Aiping Zheng

**Affiliations:** 1 State Key Laboratory of Crop Gene Exploration and Utilization in Southwest China, Sichuan Agricultural University, Chengdu 611130, China; andrewsdanso@icloud.com (A.D.O.); 13089068357@163.com (T.Z.); weileshimo@163.com (X.X.); 2020511001@stu.sicau.edu.cn (A.G.K.); irfanakbar969@gmail.com (M.I.A.); 2 Department of Plant Pathology, Rice Research Institute, Sichuan Agricultural University, Chengdu 611130, China; 3 College of Agronomy, Sichuan Agricultural University, Chengdu 611130, China; jtitriku@htu.edu.gh (J.K.T.); charlotte27appiah@gmail.com (C.A.)

**Keywords:** rice, disease resistance, qualitative resistance, quantitative resistance, R genes, gene-for-gene interactions, genome editing (CRISPR-Cas)

## Abstract

Rice (*Oryza sativa*) is a crucial staple crop for global food security, particularly in Asia. However, rice production faces significant challenges from various diseases that can cause substantial yield losses. This review explores the role of genetic resistance in rice disease management, focusing on the molecular mechanisms underlying plant–pathogen interactions and strategies for developing resistant varieties. The paper discusses qualitative and quantitative resistance, emphasizing the importance of resistance (R) genes, defense-regulator genes, and quantitative trait loci (QTLs) in conferring broad-spectrum disease resistance. Gene-for-gene relationships in rice–pathogen interactions are examined, particularly for *Xanthomonas oryzae* pv. *oryzae* and *Magnaporthe oryzae*. The review also covers recent advancements in breeding techniques, including marker-assisted selection, genetic engineering, and genome editing technologies like CRISPR-Cas. These approaches offer promising avenues for enhancing disease resistance in rice while maintaining yield potential. Understanding and exploiting genetic resistance mechanisms is crucial for developing durable and broad-spectrum disease-resistant rice varieties, essential for ensuring sustainable rice production and global food security in the face of evolving pathogen threats and changing environmental conditions.

## 1. Introduction

Rice (*Oryza sativa*) is one of the world’s most important staple crops, serving as the primary food source for over half of the global population [1]. It plays a critical role in food security, especially in Asia, which accounts for approximately 92% of the total global rice acreage [2,3]. With the global population projected to surpass eight billion by 2025, meeting the rising demand for food will require an increase in grain production by up to 50% [4]. To achieve this, reducing crop losses caused by diseases is essential. However, rice production is severely threatened by a range of diseases that can result in substantial yield losses, affecting the livelihoods of millions of farmers [5].

Among the most destructive rice diseases are rice blast, bacterial leaf blight (BLB), sheath blight, bacterial panicle blight (BPB), and rice tungro virus (Table 1). Rice blast, caused by the fungus *Pyricularia oryzae*, can infect plants at any growth stage and lead to severe yield losses, with outbreaks sometimes resulting in a total crop failure [6]. Yield losses from rice blast can range from 35% to 100%, depending on the severity of the outbreak [7]. Bacterial leaf blight, caused by *Xanthomonas oryzae*, affects rice leaves, causing yellowing and dieback, which reduces photosynthesis and grain filling, ultimately diminishing yields [8]. Sheath blight, caused by *Rhizoctonia solani*, primarily affects the leaf sheath and is especially damaging to high-yielding rice varieties. Thriving in warm, humid conditions, sheath blight can cause yield losses of up to 50% [8]. Bacterial panicle blight affects rice panicles, leading to poor grain development and lower yields [9]. Rice tungro virus, transmitted by insect vectors, causes stunted growth and yellowing of leaves, severely impacting yield [8].

These diseases not only threaten rice production but also have significant economic implications, particularly in countries where rice is a major crop [1]. To illustrate the economic and yield losses associated with these diseases, Table 1 provides a comprehensive overview of their causal agents, symptoms, geographical distribution, and economic impacts.

Effective management of rice diseases is essential to sustain production levels and ensure food security. Early detection of these diseases is critical as it allows timely interventions that can mitigate damage [21]. Traditional methods of plant disease diagnosis often rely on visual inspection and laboratory tests, which can be time-consuming and require significant expertise [22]. To mitigate the devastating effects of these diseases on rice production, it is crucial to understand the mechanisms by which plants defend themselves against pathogens.

**Figure 1:**
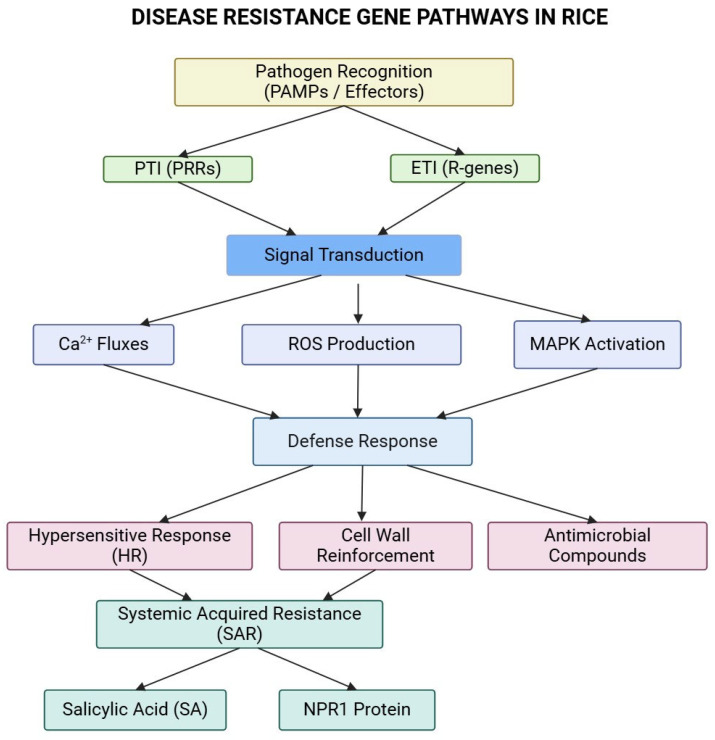
The Figure shows the signal transduction pathways involved in rice’s immune response to pathogens, highlighting the roles of PTI, ETI, and downstream defense responses.

Plants have developed a two-tiered innate immune system to protect against pathogens. The first tier is triggered when cell surface pattern recognition receptors (PRRs) detect pathogen-associated molecular patterns (PAMPs), leading to PAMP-triggered immunity (PTI) involving MAP kinase, ROS production, and defense gene regulation, influencing pathogen virulence and ETI responses [23,24] (Figure 1). However, pathogens can overcome this defense by secreting effectors that inhibit PTI, resulting in effector-triggered susceptibility (ETS) [25]. To counteract this, plants activate the second tier of defense through the expression of resistance (R) genes. These R genes specifically recognize pathogen effectors and inhibit effector-triggered immunity (ETI) to combat infection effectively [25,26]. Developing disease-resistant rice varieties is a key strategy for safeguarding food security and ensuring sustainable rice production in the face of these challenges [27].

**Figure 2:**
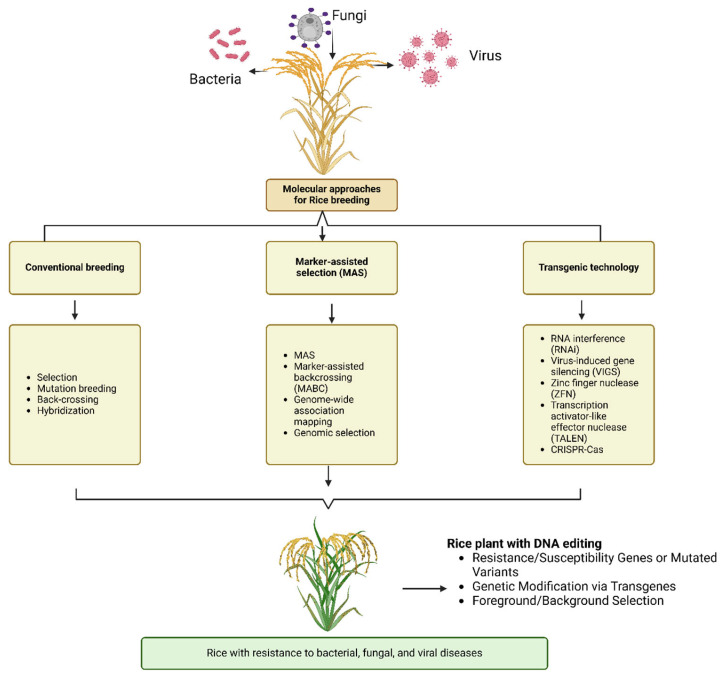
Molecular approaches for rice breeding, including conventional breeding, marker-assisted selection (MAS), and transgenic technology, to develop disease-resistant rice varieties.

Breeding disease-resistant rice varieties plays a crucial role in mitigating the impact of plant diseases on agriculture [28]. These varieties help reduce the dependence on chemical control methods, promote sustainable farming practices, and enhance food security [29]. Developing resistant rice involves identifying and incorporating resistance genes using traditional breeding [30], molecular marker-assisted selection, and genetic engineering techniques [31] (Figure 2). Key strategies include mapping disease-resistance genes at the molecular level, cloning these genes, and creating transgenic lines through genetic engineering [6,32,33,34]. While the inherent diversity of rice gene pools is a valuable resource, relying solely on natural genetic variation is insufficient for the continuous development of new resistant cultivars. To introduce further diversity, artificial mutations can be induced in the rice genome, or specific resistance-related genes can be targeted directly [32,35]. By adopting these strategies, the development of disease-resistant rice varieties will remain a cornerstone of sustainable rice production and food security.

This review explores the role of genetic resistance in rice disease management, highlighting the importance of ongoing research and innovation in developing resistant rice varieties. This review also discusses the molecular aspects of rice disease resistance, covering general methods for developing resistant plants, the genetic basis of host resistance, signal transduction pathways, defense mechanisms, the regulation of defense mechanisms by regulatory elements, and their roles in rice resistance to fungal, bacterial, and viral pathogens. Figure 2 illustrates the molecular approaches used in rice breeding, including conventional breeding, marker-assisted selection (MAS), and transgenic technology.

## 2. Genetic Resistance Mechanisms in Rice

The deployment of resistance genes in rice is crucial for disease management and environmental conservation and for reducing the dependence on chemical pesticides. Genome editing technologies, such as CRISPR-Cas, offer precise tools for targeted modifications within the rice genome, aiming to enhance the plant’s ability to combat evolving threats [36]. Recent research efforts have concentrated on identifying genes that confer broad-spectrum disease resistance in rice, such as resistance (R) genes [37], defense-regulator genes [38], and quantitative trait loci (QTLs) [39]. A genome-wide association study (GWAS) recently identified key QTLs on chromosomes 5, 6, and 9, which are linked to broad-spectrum resistance against bacterial blight. These findings offer valuable potential for the development of resistant rice varieties and the advancement of new breeding strategies [40].

Furthermore, research has also explored the role of microRNAs in modulating plant–pathogen interactions, particularly in rice–BPH interactions. It has been reported that *OsmiR319* negatively regulates resistance to brown planthopper (BPH) in rice, with its suppression resulting in enhanced resistance [41]. Additionally, *OsPCF5*, a target of *OsmiR319*, interacts with MYB proteins to mediate this resistance, emphasizing the significance of miRNA-regulated pathways in rice defense mechanisms [41]. Research has shown that miRNAs like *Osa-miR156* and *Osa-miR396* are key regulators in balancing immunity and yield in rice under biotic stresses. By managing the trade-offs between resistance and productivity, these miRNAs enable the potential assembly of important agricultural traits through genetic engineering [42]. In plants with native resistance genes, this trait is often linked to reduced yield and quality, which delays the incorporation of resistance genes into commercial varieties. A deeper understanding of genetic resistance mechanisms in rice requires distinguishing between two major types of resistance: qualitative resistance, which is governed by major genes with large effects, and quantitative resistance, which involves the combined influence of multiple genes with smaller, additive effects.

### 2.1. Qualitative Resistance

Qualitative resistance in rice is defined by distinct resistance phenotypes that follow simple Mendelian inheritance patterns, typically governed by a small number of genes with significant effects [43]. Quantitative trait loci (QTLs) are critical genetic regions linked to specific phenotypic traits, which are shaped by both genetic and environmental factors. These loci can be found on various chromosomes and are frequently used to identify potential genes responsible for particular traits [44]. These major resistance (R) genes provide a high level of protection against specific pathogen races and are easily identified and mapped [45]. This is evident in the study of Sarawak rice landraces where specific resistance genes were identified against *Pyricularia oryzae*, suggesting a qualitative resistance mechanism [43]. Operating on a gene-for-gene model, qualitative resistance involves a direct correspondence between the R gene in the rice plant and an avirulence (Avr) gene in the pathogen. This interaction can lead to a hypersensitive response, effectively halting pathogen progression [30]. However, pathogens can evolve by mutating their avirulence (Avr) genes, which can render the corresponding resistance (R) genes ineffective. This is particularly evident in the rice blast pathogen, *Magnaporthe oryzae*, where mutations in Avr genes can bypass the resistance provided by R genes such as *Pi2* and *Pi9* [30].

The genetic framework of qualitative resistance in rice primarily involves resistance (R) genes that encode receptor-like kinases (RLKs) and nucleotide-binding site-leucine-rich repeat (NLR) proteins, both of which are key components of the plant’s innate immune system [46]. RLKs are crucial for recognizing pathogen-associated molecular patterns (PAMPs), while NLRs specialize in detecting specific pathogen effectors [47,48]. This recognition activates the plant’s immune response through two distinct pathways: PAMP-triggered immunity (PTI) and effector-triggered immunity (ETI), respectively [47].

Upon pathogen recognition, these proteins initiate a series of signal transduction pathways, leading to the production of reactive oxygen species (ROS) and defense-related compounds, which inhibit pathogen proliferation [49]. NLR proteins, in particular, trigger a robust immune response, often resulting in localized cell death to prevent the pathogen from spreading. This rapid immune response is critical for preventing pathogen establishment [50]. Research has revealed that the *OsSPK1-OsRac1-RAI1* signaling pathway, utilized by distantly related NLR proteins, emphasizes the conserved mechanisms of NLR-mediated defense in rice [51].

R genes encoding receptor-like kinases (RLKs) and nucleotide-binding site-leucine-rich repeat (NLR) proteins are often clustered in the rice genome and play a crucial role in disease resistance [52]. These genes form the genetic foundation of qualitative resistance, allowing rice plants to effectively defend against pathogens. Frequently, these R gene clusters overlap with quantitative trait loci (QTLs) associated with disease resistance. For example, QTL-seq analysis has identified key genomic regions on chromosomes 1, 9, and 10 linked to resistance against dirty panicle disease, with candidate genes in these regions including RLKs and NLRs [53].

Despite its effectiveness, qualitative resistance is often race-specific, meaning it can be overcome by pathogens evolving new virulent forms [54]. For instance, *Xanthomonas oryzae* pv. *oryzae*, the bacterial blight pathogen, can evolve to bypass resistance conferred by specific R genes. In conventional agriculture, the practice of cultivating multiline cereals, each with distinct resistance to the dominant pathogen races, has been used [55]. These lines, while similar in agronomic characteristics, aim to reduce selection pressure and prevent the emergence of new pathogen variants [56]. To address this, rice breeders frequently stack multiple R genes within cultivars to improve resistance durability [57,58]. An example is the rice cultivar Zhachanglong, which contains a combination of R genes (*Xa3/Xa26*, *Xa22*(*t*), and *Xa31*(*t*)), providing broad-spectrum resistance to various strains of *Xoo* [59]. In Brazil, there is a germplasm bank with more than 20,000 rice cultivars and research is being conducted to obtain varieties resistant to multibreed *Xoo*. Wang et al. [60] reported that the engineered rice line N46(*Xa23R*), designed with stacked effector binding elements (EBEs) in the promoter region of the *xa23* gene, demonstrated broad-spectrum resistance to various strains of *Xoo* and *Xanthomonas oryzae* pv. *oryzicola* (Xoc). Importantly, this enhanced resistance was achieved without negatively impacting the plant’s growth or yield, making it a valuable development for disease management in rice breeding. Table 2 presents an overview of major qualitative resistance genes in rice, highlighting their specific roles in conferring resistance against various pathogens such as *M. oryzae* and *Xoo*. These genes play crucial roles in the plant’s defense mechanisms against rice blast and bacterial blight, two of the most devastating diseases affecting rice production worldwide.

### 2.2. Quantitative Resistance

Quantitative resistance in rice involves a complex network of multiple genetic loci, each contributing incrementally to overall disease resistance [74]. Unlike qualitative resistance, which is governed by a few major genes and offers complete or near-complete protection against specific pathogens, quantitative resistance is more durable and less susceptible to being overcome by pathogens [75]. It involves a diverse array of genes, including those involved in pathogen perception, signal transduction, and phytohormone homeostasis [74]. This type of resistance is essential for managing rice diseases as it offers a more sustainable defense approach. Key to this process is quantitative trait loci (QTLs), which play an essential role in conferring resistance to a variety of rice diseases, including rice stripe necrosis virus (RSNV) and false smut [32,45]. These QTLs, scattered across various regions of the rice genome, modulate different defense pathways to reduce the severity of infections.

Research has made significant strides in identifying and validating these QTLs, providing deeper insights into the genetic underpinnings of disease resistance. For example, the qHBV4.1 locus has been identified as a key factor in resisting rice hoja blanca disease [76]. Additionally, studies on false smut disease have uncovered “hot-spot” regions in the rice genome where disease resistance genes are concentrated. Recent research has identified several QTLs associated with resistance to rice false smut (RFS), mapped on chromosomes 2, 4, 5, 7, and 9. Among these, the novel QTL qRFSr9.1 on chromosome 9 was found to have the largest phenotypic effect, highlighting its potential importance in rice breeding programs [77]. These QTLs were linked to key resistance traits, including the number of infected panicles per plant and the total number of smut balls per panicle, both of which are critical for assessing the level of resistance [77]. In a notable study by Inoue and Hayashi [78], the mechanism of the QTL qPbm11, responsible for panicle blast resistance in the rice cultivar *Miyazaki-mochi*, was shown to operate independently from the Pb1 gene. This finding highlights the potential for enhancing rice blast resistance through the combination (or “pyramiding”) of multiple QTLs, which work together to bolster defense against *Magnaporthe oryzae*, the causative agent of rice blast.

In another key study, Zhang et al. [79] used genome-wide association analysis to identify genetic factors linked to resistance against sheath blight, a disease caused by *Rhizoctonia solani*. They found that pathways regulated by jasmonic acid (JA) and salicylic acid (SA) are crucial for mediating quantitative resistance. This discovery has significant implications for breeding programs aimed at enhancing resistance to sheath blight by focusing on these hormonal pathways. Similarly, the study by Fu et al. [80] identified a novel QTL, qRFS12.01, linked to rice false smut resistance, emphasizing the potential of quantitative resistance in breeding strategies for improved disease management. Given that no rice variety exhibits complete resistance to this pathogen, the identification of these QTLs highlights the importance of using quantitative resistance as a disease management strategy [77,81].

A novel resistance gene, identified as OsDRq12, has been located on chromosome 12 through quantitative trait locus (QTL) analysis. This gene is part of the nucleotide-binding, leucine-rich repeat (NLR) family, which plays a critical role in enhancing disease resistance in rice varieties [82]. Moreover, a comprehensive study employing pangenome-wide association studies (GWAS) has uncovered 74 QTLs linked to resistance against both panicle and leaf blast diseases. Among these, the qPBR1 locus has emerged as particularly noteworthy, demonstrating broad-spectrum resistance throughout the plant’s growth period [83]. Okello et al. [40] conducted a GWAS using a MAGIC indica panel and identified three QTLs on chromosomes 5, 6, and 9 associated with broad-spectrum resistance to African strains of bacterial blight. This study emphasizes the need for novel resistance genes to address the evolving pathogen landscape.

The cumulative integration of these QTLs, particularly those related to rice blast, sheath blight, and bacterial leaf blight, has led to the development of QTL clusters on specific chromosomal regions [84]. This method reduces the genetic intervals of QTLs and aids in identifying candidate genes that can be targeted to breed rice varieties with durable, multi-disease resistance [85]. By synthesizing these findings, researchers are making great strides in enhancing quantitative resistance in rice, ultimately paving the way for more sustainable disease management approaches in rice cultivation [86]. Table 3 presents significant quantitative trait loci (QTLs) and genes that contribute to resistance against major rice pathogens.

## 3. Gene-for-Gene Relationships in Rice–Pathogen Interaction

The gene-for-gene concept is fundamental to understanding rice–pathogen interactions, especially in the context of disease management through genetic resistance [98]. Originally introduced by Harold Flor in the 1950s [98], this theory suggests that for every resistance (R) gene in the host, there is a matching avirulence (Avr) gene in the pathogen [6]. This relationship has been extensively studied in rice, with interactions involving pathogens like *Xanthomonas oryzae* pv. *oryzae* (*Xoo*) and *Magnaporthe oryzae*, demonstrating the complexity of host–pathogen dynamics [99].

### 3.1. Gene-for-Gene Interaction in Rice and Xanthomonas oryzae

The interaction between rice and *Xanthomonas oryzae* pv. *oryzae* (*Xoo*) follows the classic gene-for-gene model where specific resistance (R) genes in rice correspond to avirulence (Avr) genes in the pathogen [100]. This gene-for-gene mechanism is crucial for rice’s immune defense, playing a central role in combating bacterial blight, a major disease impacting rice cultivation [100]. For example, R genes like *Xa23* in rice provide immunity against *Xoo* strains that express the corresponding *avrXa23* gene [60]. Similarly, other R genes, such as *Xa3*, *Xa2*, *xa5*, and *xa8*, correspond to their respective Avr genes (*avrXa3*, *avrXa2*, *avrxa5*, and *avrxa8*), contributing to disease resistance in genetically compatible rice varieties [25]. This matching system illustrates how rice can resist specific pathogen strains through these R–Avr interactions.

In this system, the recognition of Avr gene products by R genes triggers immune responses in the plant. For instance, *Xa3* can detect pathogen-associated molecular patterns (PAMPs) at the cell membrane, initiating localized immune responses [101]. This often leads to a hypersensitive response, characterized by localized cell death at the infection site to prevent pathogen spread. These interactions are mediated by the type III secretion system (T3SS) through which *Xoo* delivers Avr proteins into rice cells [60,102].

According to He et al. [103], the *AvrXa7* effector from *Xoo* binds to the promoter of the *Xa7* resistance gene in rice, activating a hypersensitive response that limits pathogen growth. This discovery underscores how specific R–Avr interactions can be leveraged to develop broad-spectrum resistance strategies in rice. Supporting this, research by Zou et al. [104] emphasized that the recognition of Avr genes triggers defense mechanisms in rice, effectively halting pathogen development and disease progression. These insights highlight the critical role of R–Avr interactions in managing bacterial blight.

The gene-for-gene interaction between rice and *Xoo* involves a complex interplay between rice’s R genes and pathogen effectors, primarily the transcription activator-like effectors (TALEs) produced by *X. oryzae* [105]. TALEs are secreted into rice cells via the T3SS where they act as transcription factors, activating susceptibility genes in rice or suppressing immune responses [58]. However, many R genes in rice have evolved to recognize these TALEs and trigger defense mechanisms to prevent disease progression [105]. For example, R genes like Xa1, Xa10, and Xa23 detect TALEs and initiate a hypersensitive response, limiting the pathogen’s spread [58,60].

In some instances, truncated versions of TALEs, known as interfering TALEs (iTALEs), produced by *Xoo* can interfere with this recognition process [106]. These iTALEs can suppress the resistance response mediated by R genes such as Xa1, enabling the pathogen to evade host detection [107]. This dynamic co-evolution between rice and *Xoo* highlights a continuous arms race, with the pathogen evolving strategies to evade recognition, while rice evolves to recognize and counter these pathogenic effectors [108].

The avrBs3/pthA family of Avr genes in *Xoo* has been extensively studied for its role in triggering resistance responses in rice. This gene family is diverse, occurring individually or in clusters within the pathogen’s genome [109]. The gene-for-gene model explains why some rice cultivars are resistant to bacterial blight while others remain susceptible. Additionally, it reveals that certain R genes share signaling components. For example, *Xa3* shares defense signaling pathways with *Xa21*, suggesting overlapping defense mechanisms while maintaining distinct roles in pathogen recognition [110].

Several R genes, such as *Xa3*, *Xa26,* and *Xa4,* encode receptor-like kinases that confer resistance to *Xoo*. These receptor-like kinases detect PAMPs and initiate defense responses, including cell wall strengthening and activation of downstream signaling pathways that result in resistance [58]. The ability of these receptor-like kinases to detect pathogen molecules and initiate defense responses is a key aspect of rice’s immune system.

Understanding these gene-for-gene interactions provides valuable insights into breeding rice cultivars with durable resistance to bacterial blight. Identifying both R genes in rice and Avr genes in *Xoo* is critical for developing effective disease management strategies. This knowledge supports the development of rice varieties capable of withstanding pathogen attacks, promoting sustainable agricultural practices, and enhancing global food security.

### 3.2. Gene-for-Gene Interaction in Rice and Magnaporthe oryzae

The gene-for-gene interaction in the rice-*Magnaporthe oryzae* pathosystem exemplifies a highly specialized co-evolutionary relationship. In this interaction, specific rice resistance (R) genes, such as Pi genes including *Pi-ta*, *Pia*, and *Pii*, recognize corresponding avirulence (Avr) genes in *M. oryzae*, providing resistance against pathogen strains that express these genes [6]. For instance, the *Pi-ta* gene can detect the *AVR-Pita* effector, triggering a hypersensitive response (HR) that inhibits pathogen growth [30,111]. This interaction, beyond providing immediate defense, also drives ongoing genetic adaptation in rice as pathogens evolve new Avr gene variants to circumvent existing resistance [112]. Research on *AvrPi54* and *AvrPii* has shown that these Avr genes are subject to mutation and haplotype diversification, which allows pathogens to evade detection by R genes in rice, challenging the crop’s defensive capacity [113]. The emergence of new Avr gene variants, like those in *AVR-Pi9*, illustrates the relentless genetic “arms race” between rice and its pathogens. This dynamic underscores the necessity of consistent monitoring and adaptive strategies in rice breeding programs to address these evolving threats [114].

Extensive research has characterized over 30 rice R genes and 12 Avr genes in *M. oryzae*, documenting their interactions [115]. These gene-for-gene relationships are mediated by nucleotide-binding leucine-rich repeat (NLR) proteins encoded by R genes, which act as receptors recognizing pathogen effectors and triggering HR—a form of programmed cell death that limits pathogen spread. The *Pi-ta*/*AVR-Pita* interaction serves as a model where the cytoplasmic NLR protein encoded by *Pi-ta* interacts directly with *AVR-Pita*, triggering defense responses [116]. Other well-studied R–Avr pairs, such as *Pi54/AVR-Pi54*, *Pik/AVR-Pik*, and *Pia/AVR-Pia*, have furthered our understanding of direct and indirect molecular recognition mechanisms [117].

The identification of new Avr genes, like *AVR-Mgk1*, underscores the persistent co-evolution between rice and *M. oryzae*, providing insights into the adaptive strategies employed by both host and pathogen [118]. These findings support the development of novel resistance approaches that can address the pathogen’s evolving threat [118]. Additionally, rice’s defensive capabilities are enhanced by the presence of multiple R genes, which together provide broad-spectrum resistance against diverse *M. oryzae* strains [115], Thus underlining the importance of understanding these genetic distinctions in rice and its pathogens to devise effective disease management practices as certain strains may exhibit varying levels of adaptation to different rice subspecies, such as indica and japonica [119].

### 3.3. Gene-for-Gene Interaction in Rice and Brown Planthopper (BPH)

A gene-for-gene interaction model is similarly observed in rice’s response to the brown planthopper (BPH), *Nilaparvata lugens*, which is a significant pest in rice agriculture. Researchers have identified multiple resistance (R) genes, including *Bph14*, *Bph17*, and *Bph32*, that confer resistance to various BPH populations by recognizing distinct avirulence (Avr) gene profiles in the pest [120,121,122]. These R genes are located in specific regions of the rice genome and trigger a cascade of defense responses upon detecting their corresponding Avr genes [123]. The resistance gene *Bph14* is particularly noteworthy as it can confer resistance to specific BPH biotypes [124,125]. However, virulent strains of BPH have developed mechanisms to overcome this resistance. Upon activation, Bph14 not only leads to rapid callose deposition and lignin accumulation but also triggers the synthesis of various phytochemical compounds that enhance the plant’s defensive capabilities against BPH attack. These compounds contribute to strengthening physical barriers that make it more challenging for BPH to penetrate the tissue [126,127]. Similarly, *Bph17* and *Bph32* activate comparable defensive pathways, each providing resistance against different BPH biotypes and, thereby, enhancing the plant’s defense arsenal [122]. This interaction mirrors the evolutionary dynamics observed in the rice—*M. oryzae* pathosystem where genetic variability in both host and pest populations drives changes in virulence allele frequencies [128]. This underscores the importance of ongoing adaptation in crop protection.

## 4. Molecular Mechanisms Underlying Gene-for-Gene Interactions

Mitogen-activated protein kinase (MAPK) cascades play a pivotal role in mediating plant defense responses in rice against Xoo infection. These signaling pathways are activated upon pathogen recognition, leading to a series of phosphorylation events that enhance the plant’s immune response [129]. Transcriptome-based analyses have highlighted the critical role of these MAPK cascades in regulating immune responses, including the reinforcement of cell wall barriers and the production of antimicrobial compounds [130]. Specific MAPK components, such as OsMKK6 and OsMPK4, form a signaling cascade that regulates immune responses and improves resistance to bacterial blight caused by Xoo [129]. In addition to MAPK signaling, transcription factors like WRKY13 and WRKY45 also play crucial roles in integrating both biotic and abiotic stress signals, modulating gene expression patterns that facilitate both localized and systemic defenses. These transcription factors work in concert with MAPK signaling to coordinate various defense responses [131,132].

The rapid induction of MAPK genes such as OsMPK3, OsMPK4, and OsMPK6 occurs within hours of Xoo infection, indicating their role in early defense signaling [133]. The activation of these MAPKs triggers downstream transcription factors like OsWRKY45, which are essential for orchestrating defense gene expression [134,135]. Moreover, plants also form physical barriers, such as callose deposition and lignin synthesis, contributing to the structural reinforcement of cell walls and preventing further pathogen invasion [136]. This interplay between biochemical signaling and physical defenses underpins the overall resistance mechanisms in rice [137].

The relationship between MAPKs and pathogen effectors is complex, with the pathogen using various strategies to undermine plant defenses [138]. For example, non-TAL effectors from Xoo can inhibit MAPK activation, illustrating the pathogen’s ability to suppress the plant’s immune system. Overall, the elucidation of MAPK cascades in rice provides valuable insights into the molecular mechanisms underlying disease resistance and offers potential targets for enhancing resistance through genetic engineering or breeding strategies.

## 5. Breeding for Disease-Resistant Varieties

### 5.1. Conventional Breeding Methods

Conventional breeding techniques have long been a cornerstone in the development of rice varieties resistant to major diseases that threaten crop yields and food security. Traditional methods such as selection, hybridization, and backcrossing have been successfully used to incorporate resistance to critical pathogens, including those responsible for blast, bacterial blight, and sheath blight [139,140]. Breeders focus on transferring resistance genes from wild or resistant rice varieties into high-yielding but susceptible commercial cultivars. For example, Pi2 and Pi9, genes conferring resistance to rice blast, have been integrated into cultivated varieties to counter the fungal pathogen *M. oryzae* [140,141].

However, conventional breeding faces challenges, particularly the issue of linkage drag where undesirable traits are unintentionally co-selected alongside the beneficial resistance genes. This can lead to reductions in other important agronomic characteristics, such as grain yield or quality, making the resulting varieties less desirable for farmers [142]. Moreover, the process is time-consuming and labor-intensive, often requiring multiple generations of crossing and backcrossing to achieve the desired resistance traits alongside favorable agronomic qualities [143]. Additionally, resistance derived from conventional breeding may not be long-lasting due to the ability of pathogens to adapt, resulting in resistance breakdown over time [144].

A significant limitation of conventional breeding is the trade-off between disease resistance and other key traits. For example, the introduction of resistance genes might come at the cost of reduced yield or quality if linkage drag occurs [145]. As a result, breeders often face difficult decisions in balancing disease resistance with the maintenance of high yield and other desirable agronomic traits [146]. These trade-offs represent ongoing challenges in conventional breeding methods. Further improvements in breeding efficiency and the management of genetic trade-offs will be vital for advancing rice disease resistance through traditional methods.

To address some of these limitations, marker-assisted selection (MAS) has emerged as a powerful tool that complements conventional breeding by improving the precision and efficiency of the breeding process.

### 5.2. Marker-Assisted Selection

Marker-assisted selection (MAS) plays a crucial role in modern rice breeding by allowing for the precise incorporation of resistance genes into rice cultivars, thereby improving their resilience against various pathogens [147]. This technique has been essential for developing rice varieties with enhanced resistance to diseases such as rice blast and bacterial blight, which pose significant threats to global rice production. MAS enables the identification and pyramiding of multiple resistance genes, resulting in the creation of rice lines with broad-spectrum and durable resistance [35]. However, developing MAS technology is difficult in emerging countries because it requires money, equipment, and trained personnel

One major success of MAS in rice breeding is its contribution to improving resistance to rice blast and brown planthoppers. In China, MAS has been used to develop rice lines such as Huahui7713 and Huahui3006, which incorporate resistance genes *Pigm*, *Bph6*, and *Bph9*. These lines have been utilized to create hybrid varieties like Weiliangyou7713 and Xuanliangyou3006, which demonstrate enhanced resistance to both rice blast and brown planthoppers while maintaining high yields and quality [35]. These varieties have been widely cultivated, reducing the need for chemical controls. Similarly, MAS has been used in India to introgress resistance genes Xa21, xa13, and xa5 into the aromatic rice cultivar HUR 917 [148]. This has led to the development of near-isogenic lines that exhibit broad-spectrum resistance to bacterial blight while preserving the desirable traits of the parent cultivar, thereby contributing to sustainable rice production [148]. In Jiangsu Province, China, MAS was used to successfully introduce the *Pigm* gene into rice cultivars. This gene provides durable and broad-spectrum resistance to rice blast without negatively affecting yield or other agronomic traits, leading to the development of the rice line Yangnonggeng 3091, which has been recognized for its excellent performance in yield, quality, and disease resistance [149].

Despite these successes, MAS faces several limitations. One significant challenge is the rapid evolution of pathogen strains, which can overcome the resistance conferred by single-resistance genes [70]. For example, the *Xa23* gene, which provides resistance to bacterial blight, has been overcome by new *Xoo* isolates. This underscores the need for continuous monitoring and the development of strategies that involve stacking multiple effector binding elements (EBEs) to broaden resistance [60]. Another limitation of MAS is the complexity of gene pyramiding, which involves combining multiple resistance genes into a single cultivar. This process is influenced by factors such as the number of genes involved, genetic background, and environmental interactions, making it a complex and time-consuming task [150]. Moreover, MAS often targets specific pathogens or strains, which may not offer comprehensive protection against all potential threats. For instance, while MAS has been successful in developing resistance to certain strains of bacterial blight and blast, it may not be as effective against other emerging diseases or pests [151].

While MAS has made significant contributions to the development of resistant rice cultivars, its effectiveness can be challenged by the rapid evolution of pathogens and the complexity of gene interactions [152]. To overcome these challenges, continuous innovation in breeding strategies, including the integration of genome editing and high-throughput phenotyping, is essential to ensure the sustainability of rice production amidst evolving biotic stresses [153,154]. The combination of genetic improvements with IPM practices offers a promising path forward for sustainable rice production [155]. However, the challenges in widespread adoption and the need for region-specific adaptations highlight the complexity of implementing MAS in diverse agricultural systems [156]. While marker-assisted selection has been a valuable tool for rice improvement over the past few decades, recent advances in genome editing technologies like CRISPR/Cas9 are now revolutionizing rice breeding by allowing precise modifications directly in elite cultivars [157].

### 5.3. CRISPR/Cas9 and Gene Editing in Rice

**Figure 3:**
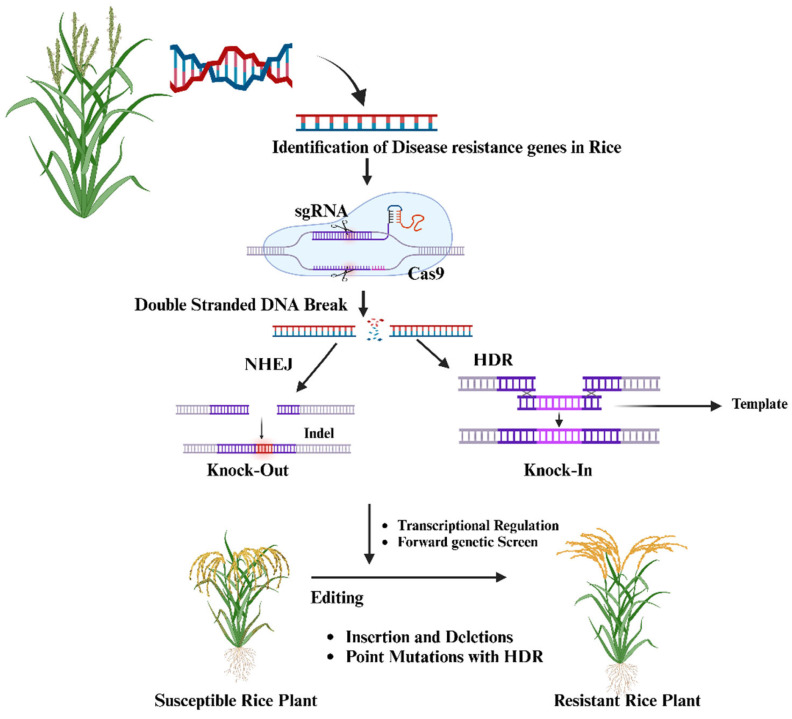
This Figure illustrates the CRISPR-Cas9 editing process used to create disease-resistant rice plants by knocking out or modifying disease-susceptibility genes.

CRISPR/Cas9 technology has emerged as a transformative tool for enhancing disease resistance in rice, with a particular focus on combating bacterial blight caused by *Xanthomonas oryzae* pv. *oryzae* (*Xoo*) [60]. By targeting specific genes associated with disease susceptibility, researchers can introduce precise edits that either knock out or modify these genes, leading to improved resistance. The process involves designing a single-guide RNA (sgRNA) to direct the Cas9 protein to the target DNA sequence where it creates a double-stranded break [158]. This break is then repaired through either non-homologous end joining (NHEJ), which introduces indels that knock out the gene, or homology-directed repair (HDR) [158], which allows for precise modifications using a template (Figure 3). These edits result in rice plants with enhanced resistance to diseases such as bacterial leaf blight and rice blast [159]. By targeting susceptibility genes in rice, such as *OsSWEET14*, which *Xoo* exploits using transcription activator-like effectors (TALEs), CRISPR/Cas9 enables the modification of these vulnerable genetic sites [159]. These TALEs bind to specific promoter regions in *OsSWEET14*, facilitating infection and allowing the pathogen to redirect plant nutrients for its growth [160]. Research on Super Basmati rice employed CRISPR/Cas9 to disrupt the effector binding elements (EBEs) within the *OsSWEET14* promoter, which TALEs like *AvrXa7*, *PthXo3*, and TalF recognize. By modifying these EBEs, scientists developed rice lines (SB-E1, SB-E2, SB-E3, and SB-E4) that are resistant to *Xoo*, thus preventing TALE binding and reducing bacterial blight incidence [161]. This resistance was validated by significantly shorter lesion lengths in edited plants relative to unmodified controls [162]. Thus, CRISPR/Cas9 not only enhances rice’s resilience to bacterial blight but also provides a method for creating disease-resistant rice varieties without introducing foreign genes, making it a valuable, sustainable tool for agriculture [163,164].

Beyond bacterial blight, CRISPR/Cas9 has been successfully applied to address a wider spectrum of bacterial and fungal diseases in rice [165]. Through modifications to the *xa23* gene promoter, researchers introduced multiple EBEs responsive to various strains of *Xanthomonas oryzae*, providing broad-spectrum resistance to both bacterial blight and bacterial leaf streak without impacting plant growth [60]. Further work in transcription factor mutagenesis demonstrated that editing NAC transcription factor genes with CRISPR/Cas9 can enhance rice’s natural immunity to microbial pathogens, with the added advantage of maintaining crop yield [166].

The technology has also proven effective in combating fungal diseases, notably rice blast. Knocking out the *Bsr-d1* gene, a susceptibility gene for rice blast, has been shown to significantly enhance resistance to this common fungal threat, particularly in japonica rice where gene modification improved resistance from the seedling stage onward [167]. In addition, CRISPR/Cas9 has been used for simultaneous editing of multiple genes, such as *Pi21* and *OsSULTR3;6*, thereby conferring resistance to both rice blast and bacterial leaf streak while preserving agronomic traits [167]. This capability highlights CRISPR/Cas9’s potential to provide resistance to multiple pathogens without trade-offs in plant performance.

The technology’s scope also extends to modifying defense pathways for broad-spectrum disease resistance. For instance, CRISPR/Cas9-mediated mutation of the *OsS5H* genes, which are part of the salicylic acid pathway, has resulted in rice plants with enhanced resistance to diverse pathogens. Triple mutants of the *OsS5H* gene showed strong resistance to both bacterial blight and rice blast due to upregulated defense-related genes, underscoring the value of targeting systemic resistance pathways [168].

Finally, combining CRISPR/Cas9 with traditional breeding and other advanced biotechnological approaches holds significant promise for the development of high-yielding, resilient rice varieties. This integration aims to enhance productivity and sustainability in rice cultivation, addressing the pressing global need for resilient food crops in an increasingly challenging agricultural landscape [163].

## 6. Disease-Resistant Rice Varieties and Their Impact on Disease Management

Disease-resistant rice varieties have been developed to counter major rice pathogens. These varieties incorporate resistance genes or traits that enable the plant to defend itself against specific pathogens, reducing the need for chemical interventions and improving overall crop health and productivity [169]. By examining specific examples of disease-resistant rice varieties and their effects on disease management, we can better understand the importance of this approach in modern agriculture. Table 4 highlights key rice varieties incorporating disease resistance genes, demonstrating their significant contributions to managing major rice diseases such as bacterial blight and rice blast. These varieties showcase the practical application of genetic resistance in rice breeding programs

## 7. Environmental Impact of Disease-Resistant Rice Varieties

Developing rice varieties that are resistant to disease has become a valuable agricultural approach to lessening environmental damage, mainly by reducing the need for pesticides [8,146]. These rice strains are specifically crafted to resist diseases like bacterial leaf blight and blast, which are frequent threats to rice crops. With inherent disease resistance, farmers can rely less on chemical pesticides, which are typically applied in large quantities, to manage crop diseases. Reducing pesticide use not only lowers production costs for farmers but also greatly decreases the chance of pesticide runoff into nearby waterways [35]. Less pesticide runoff means lower levels of soil and water pollution, thus helping preserve the quality of these resources and fostering a healthier ecosystem for surrounding plants and animals [181].

Additionally, using fewer pesticides helps protect biodiversity in rice-farming areas [182]. Pesticides often harm non-target organisms, including helpful insects, soil microorganisms, and aquatic life in nearby water bodies [182]. Disease-resistant rice varieties, by reducing the need for chemical applications, support the survival of these beneficial species, promoting a balanced and diverse ecosystem [183]. Maintaining biodiversity is crucial as it contributes to natural pest control, soil health, and overall resilience in agricultural environments [184]. Ecosystems rich in biodiversity are generally more stable and adaptable to environmental changes, offering long-term advantages for sustainable farming [185].

However, cultivating disease-resistant rice varieties may pose certain ecological risks. One of these is genetic contamination where genes from genetically modified or selectively bred disease-resistant rice could transfer to wild rice populations through cross-pollination [186]. This gene flow might change the genetic structure of wild rice species, potentially affecting their natural disease resistance, growth, or ability to adapt to native conditions [187]. Moreover, the spread of resistant genes into wild populations could decrease the genetic diversity of wild rice, which is vital for their resilience against climate change and other environmental pressures [188]. There are also concerns about the broader ecological impact if these resistant traits become dominant as this could upset the balance of ecosystems that include wild rice species [189].

In summary, cultivating disease-resistant rice varieties offers significant environmental advantages, particularly by lowering chemical pesticide use, safeguarding water and soil quality, and promoting biodiversity. However, careful oversight is essential to minimize risks like genetic contamination and unintended consequences for wild rice species. Overall, disease-resistant rice represents a promising step toward more sustainable agriculture with an emphasis on environmental responsibility.

## 8. Future Perspectives and Challenges

As the global demand for rice continues to rise, the development of disease-resistant varieties becomes increasingly critical to ensuring food security. Looking ahead, the field faces both exciting opportunities and complex challenges.

Advances in gene editing technologies, particularly CRISPR-Cas, provide unprecedented precision for modifying rice genomes to enhance disease resistance. These tools enable the targeted alteration of susceptibility genes or the introduction of novel resistance mechanisms. However, the widespread application of these technologies still faces challenges, including optimizing their efficiency and addressing regulatory concerns across different regions.

With the ongoing impacts of climate change, rice pathogens are expected to evolve and spread to new geographical areas. Consequently, breeding efforts will need to focus not only on improving disease resistance but also on developing varieties that are adaptable to shifting environmental conditions. This will require an integrated approach that combines disease resistance with traits for tolerance to abiotic stresses such as drought, salinity, and heat.

As pathogens continue to evolve, ensuring the durability of disease resistance remains a significant challenge. Future research will need to prioritize strategies for creating long-lasting resistance, such as pyramiding multiple resistance genes to provide broader protection, exploring quantitative trait loci (QTLs) that offer partial but more stable resistance, and identifying new sources of resistance from wild rice species.

The integration of multi-omics technologies, including genomics, transcriptomics, proteomics, and metabolomics, will be key to gaining a deeper understanding of rice–pathogen interactions. This comprehensive approach could lead to the discovery of novel resistance mechanisms and potential targets for breeding, ultimately making breeding programs more effective.

Future breeding efforts must also align with sustainable agriculture practices, which include reducing reliance on chemical inputs and ensuring compatibility with integrated pest management strategies. Balancing high yield potential with robust disease resistance presents an ongoing challenge in creating varieties that are both productive and environmentally friendly.

Finally, as new breeding technologies emerge, addressing public concerns and navigating regulatory frameworks will be crucial. Ensuring transparency in the development and deployment of these technologies, along with engaging the public in dialogue, will be essential to gaining acceptance and ensuring the widespread adoption of disease-resistant rice varieties.

## 9. Conclusions

The development of disease-resistant rice varieties is a rapidly evolving field, driven by advancements in genetic and molecular technologies. The gene-for-gene model has laid the groundwork for understanding plant–pathogen interactions, shaping breeding strategies aimed at improving resistance to major rice diseases. Significant strides have been made in identifying and characterizing key resistance genes, such as those responsible for conferring resistance to bacterial blight (Xa genes) and blast (Pi genes). These breakthroughs have enabled more precise and targeted breeding methods, including marker-assisted selection and gene pyramiding, which have led to the creation of rice varieties with enhanced and more comprehensive disease resistance. The advent of cutting-edge technologies, particularly genome editing tools like CRISPR-Cas, has opened up new possibilities for making precise genetic modifications to further boost disease resistance. These innovations, along with a deeper understanding of the molecular mechanisms behind plant immunity, promise to make breeding efforts more efficient and effective. Despite these advances, several challenges persist. The continuous evolution of pathogens, the complex nature of quantitative resistance, and the need to balance disease resistance with other critical agronomic traits remain significant obstacles. Additionally, the impacts of climate change on pathogen dynamics introduce further complications for breeders. Looking ahead, a comprehensive approach combining conventional breeding, molecular techniques, and emerging technologies will be essential for developing durable and broad-spectrum disease resistance in rice. This integrated strategy, alongside sustainable agricultural practices, will play a crucial role in ensuring global food security in the face of evolving pathogen threats and shifting environmental conditions. The ongoing collaboration between plant breeders, pathologists, molecular biologists, and agronomists will be instrumental in overcoming these challenges and ensuring the long-term resilience of rice as a staple crop.

## Figures and Tables

**Table 1:** Overview of rice diseases, their causal agents, symptoms, geographical distribution, and economic impact.

Disease	Causal Agent	Symptoms	Geographical Location	Economic Impact	References
Rice blast	*Magnaporthe oryzae*	Leaf spots, neck rot, reduced grain filling	Asia, Africa, Latin America	Significant yield losses up to 50% in severe cases	[10,11]
Bacterial blight	*Xanthomonas oryzae* pv. *oryzae*	Yellowing of leaves, wilting, reduced grain quality	Southeast Asia, South Asia, Africa	Reduced yield, potential loss of up to 70% in vulnerable areas	[12,13]
Sheath blight	*Rhizoctonia solani*	Sheath lesions, lodging, poor grain quality	Asia, Americas, Africa	Yield reduction by 20–50%, increased cost for fungicides	[14]
Rice tungro disease	Rice tungro viruses (RTSV, RTBV)	Stunting, yellow-orange leaf discoloration	South and Southeast Asia	Yield losses up to 100% in severe outbreaks	[15,16]
Brown spot	*Bipolaris oryzae*	Brown lesions on leaves, reduced grain filling	Worldwide, especially in South Asia	Up to 90% yield loss in epidermic conditions	[17,18]
Bakanae disease	*Fusarium fujikuroi* *F. proliferatum* *F. verticillioides* *F. andiyazi*	Wilting of rice plant, yellowing of leaves (Chlorosis), root decay, reduced tillering	Southeast Asia, sub-Saharan Africa, South Asia and Latin America	Losses, ranging from 3.0% to 95.4%, depending on the regions and varieties grown	[19,20]

**Table 2:** Major qualitative resistance genes in rice and their roles in disease resistance.

Genes	Role	References
*Pi-ta*	Provides resistance against the rice blast fungus, *Magnaporthe oryzae*	[61,62]
Xa21	Confers resistance to bacterial blight caused by *Xoo*	[63,64]
*Pi-b*	Offers resistance to specific strains of the rice blast pathogen	[65,66]
*Pi-kh*	Another gene associated with resistance to rice blast disease	[39,67]
*Xa5*	Provides resistance to bacterial blight, particularly effective against specific races of the pathogen	[68,69]
*Xa13*	Confers resistance to bacterial blight and is known for its effectiveness against certain pathogen strains	[70]
*Pi-2*	Offers resistance to rice blast and is associated with a broader spectrum of pathogen races	[71,72]
*Pi-1*	Provides resistance against *Magnaporthe oryzae* and is commonly used in breeding programs	[73]
*Xa23*	Functions by trapping specific *Xoo* type III secreted effectors, activating a hypersensitive response (HR) in the host plant	[60]
*Xa7*	Interacts with *Xoo* effectors, contributing to the plant’s defense mechanisms	[70]

**Table 3:** Key quantitative trait loci (QTLs) and genes conferring resistance to major rice pathogens.

QTL/Gene	Role in Resistance	Pathogen Targeted	References
*hb9-2*	Provides quantitative resistance to sheath blight	*R. solani*	[87]
*Pi21*	Offers partial resistance to blast disease	*M. oryzae*	[88,89]
*qBR11-1*	Enhances broad-spectrum resistance to bacterial blight	*X. oryzae* pv. *oryzae*	[90,91]
*qSBR11*	Contributes to resistance against sheath blight	*R. solani*	[92]
*qSB-9*	Reduces disease severity in sheath blight	*R. solani*	[93,94]
*Pi35*	Confers quantitative resistance to blast disease	*M. oryzae*	[88,95]
*qBlsr5a*	Enhances resistance to bacterial leaf streak	*Xanthomonas oryzae* pv. *oryzicola*	[96,97]

**Table 4:** Notable disease-resistant rice varieties and their impact on disease management.

Variety	Resistant Gene	Target Disease	Impact on Disease Management	References
IR36	*X4*, *X5*, *X13*	Bacterial blight	Provided broad-spectrum resistance to multiple races of bacterial blight pathogens. Widely adopted across Asia, stabilizing yields.	[170]
IR64	*Xa4, Xa5*	Bacterial blight	Improved resistance to bacterial blight. Widely grown variety in Asia.	[171]
IRBB21	*Xa21*	Bacterial blight	Conferred strong, broad-spectrum resistance to bacterial blight. Used as a donor in breeding programs.	[172,173]
Utyos	Derived from IR-36	Rice Blast	High resistance to blast and high yield potential. Widely grown in Russia.	[174]
Puta Basmati 1509	*Pi2, Pi54*	Rice blast	First blast-resistant Basmati variety. Reduced fungicide use in Basmati growing regions.	[67,175]
C101A51	*Pi-1, Pi-2, Pi-33*	Rice blast	Pyramided multiple blast resistance genes. Used as a donor in breeding programs.	[176,177]
Super 1000	*Xa23, Pi9*	Bacterial blight, Rice blast	Enhanced disease resistance while maintaining high yield, showcasing a model for developing disease-resistant rice varieties.	[178]
IR72	*Xa21*	Bacterial Blight	Improved resistance to *Xoo.*	[179]
Minghui 63	*Xa21, Xa23*	Bacterial blight	Restorer line with pyramided bacterial blight resistance. Used in hybrid rice breeding.	[180]

## Data Availability

No new data were created or analyzed in this study.
